# Multi-Regional Modeling of Cumulative COVID-19 Cases Integrated with Environmental Forest Knowledge Estimation: A Deep Learning Ensemble Approach

**DOI:** 10.3390/ijerph19020738

**Published:** 2022-01-10

**Authors:** Abdelgader Alamrouni, Fidan Aslanova, Sagiru Mati, Hamza Sabo Maccido, Afaf. A. Jibril, A. G. Usman, S. I. Abba

**Affiliations:** 1 Department of Environmental Education and Management, Faculty of Education, Near East University, Nicosia 700006, Cyprus; 20167251@std.neu.edu.tr; 2 Department of Environmental Engineering, Faculty of Civil and Environmental Engineering, Near East University, Nicosia 700006, Cyprus; fidan.aslanova@neu.edu.tr; 3 Department of Economics, Yusuf Maitama Sule University, Kano 700282, Nigeria; smati@smati.com.ng; 4 Department of Electrical and Computer Engineering, Faculty of Engineering, Baze University, Abuja 900288, Nigeria; hamza.maccido@bazeuniversity.edu.ng; 5 Faculty of Clinical Sciences, Bayero University, Kano 700006, Nigeria; afafabubakar@gmail.com; 6 Department of Analytical Chemistry, Faculty of Pharmacy, Near East University, TRNC, Mersin 99138, Turkey; abdullahigusman@gmail.com; 7 Interdisciplinary Research Center for Membrane and Water Security, King Fahd University of Petroleum and Minerals, Dhahran 31261, Saudi Arabia; 8 Department of Civil Engineering, Faculty of Engineering, Baze University, Abuja 900288, Nigeria

**Keywords:** artificial intelligence, ARIMA, ensemble ARIMA, forest knowledge, prediction

## Abstract

Reliable modeling of novel commutative cases of COVID-19 (CCC) is essential for determining hospitalization needs and providing the benchmark for health-related policies. The current study proposes multi-regional modeling of CCC cases for the first scenario using autoregressive integrated moving average (ARIMA) based on automatic routines (AUTOARIMA), ARIMA with maximum likelihood (ARIMAML), and ARIMA with generalized least squares method (ARIMAGLS) and ensembled (ARIMAML-ARIMAGLS). Subsequently, different deep learning (DL) models viz: long short-term memory (LSTM), random forest (RF), and ensemble learning (EML) were applied to the second scenario to predict the effect of forest knowledge (FK) during the COVID-19 pandemic. For this purpose, augmented Dickey–Fuller (ADF) and Phillips–Perron (PP) unit root tests, autocorrelation function (ACF), partial autocorrelation function (PACF), Schwarz information criterion (SIC), and residual diagnostics were considered in determining the best ARIMA model for cumulative COVID-19 cases (CCC) across multi-region countries. Seven different performance criteria were used to evaluate the accuracy of the models. The obtained results justified both types of ARIMA model, with ARIMAGLS and ensemble ARIMA demonstrating superiority to the other models. Among the DL models analyzed, LSTM-M1 emerged as the best and most reliable estimation model, with both RF and LSTM attaining more than 80% prediction accuracy. While the EML of the DL proved merit with 96% accuracy. The outcomes of the two scenarios indicate the superiority of ARIMA time series and DL models in further decision making for FK.

## 1. Introduction

On 31 December 2019, there were many instances of pneumonia in China with no known background. The cases were reported in early December 2019, and many of those who were infected lived or worked at the Huanan local Seafood Wholesale Market, despite the fact that the remainder of the cases had no connection to this location [1]. A novel coronavirus, designated as 2019-nCoV by WHO on 7 January, was discovered in one of these patients [2]. The new virus was termed severe acute respiratory syndrome coronavirus 2 (SARS-CoV-2) by the Coronavirus Study Team (CST) [2], and was later renamed COVID-19 by WHO. As of 30 January 2020, seven thousand, seven hundred and thirty-six verified incidents and twelve thousand, one hundred and sixty-seven probable occurrences had been reported in the Republic of China, with eighty-two instances reported in eighteen additional countries [3]. On 30 January 2020, WHO designated the SARS-CoV-2 epidemic a public health emergency of international concern (PHEI) and a pandemic on 11 March 2020 [3,4].

According to the Chinese National Health Commission, the percentage of deaths among confirmed cases in the Republic of China was 2.1% as of 4 February 2020 [4]. COVID-19 spreads quickly, resulting in a high number of deaths; moreover, accessible data as well as published findings are rapidly expanding. As of 17 May 2021, the disease had claimed the lives of over three million individuals, with over 163 million confirmed cases in over 220 nations and territories [5]. It is unclear what influence the Huanan Seafood Market played in the transmission of the new virus. The majority of the first COVID-19 cases were related to this market, indicating that the virus was likely transmitted from animals to humans [6]. According to genetic evidence, the virus was introduced into the Huanan market from an unknown source and quickly spread throughout the city, despite the fact that human-to-human transmission is known to have occurred earlier [6].

Human-to-human transmission was first suggested by the large number of affected family members and later confirmed by health experts [6]. COVID-19 has had a detrimental influence on Africa’s health, security, politics, and society. Already frail healthcare facilities were overwhelmed by the rapidly growing number of cases during the pandemic’s dramatic peak. The continued function of vital health services has also been disturbed in several African nations, resulting in a supply–demand imbalance. The pandemic has had a significant impact on non-communicable disease treatment, regular vaccination, prenatal care, family planning and contraception, and other services. Several researchers have attempted to anticipate the CCC [7,8], with ARIMA being used in several of these studies [9,10]. Seasonal ARIMA for example, SARIMA model is associated with epidemiological models based on phone call data [11,12]. Some research, on the other hand, focused on the impact of governmental measures—such as lockdown and social separation—on COVID-19 dissemination [11].

**Figure 1:**
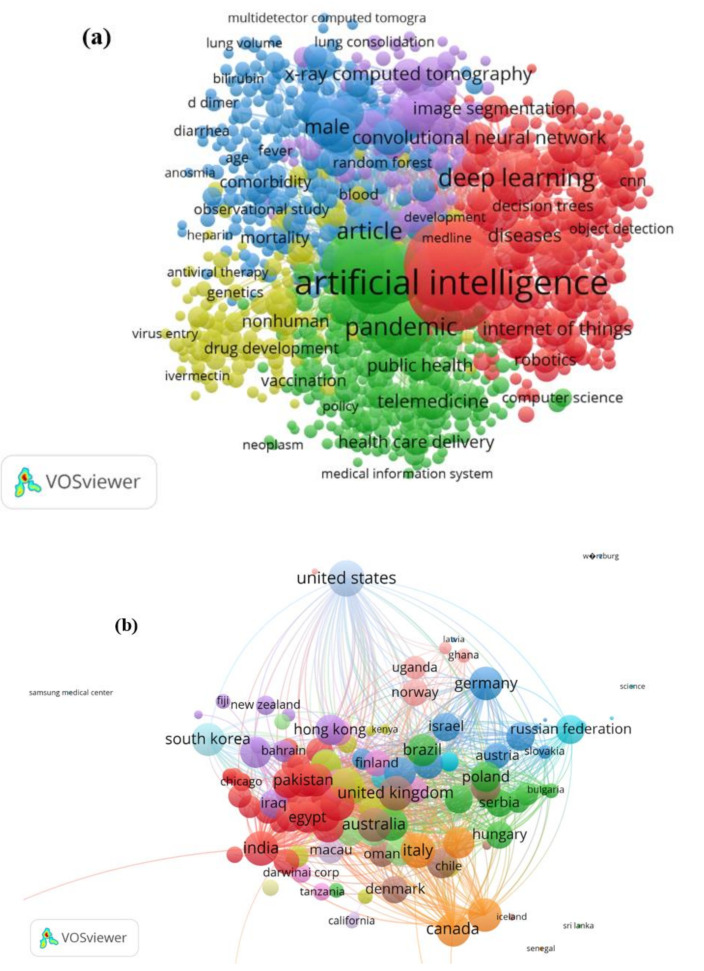
(**a**) Major keywords in the literature on COVID-19, determined using machine learning models (2020–2021); (**b**) investigated research regions for the COVID-19 prediction.

Various new AI-based models have yet to be applied to COVID-19 situations, despite suggestions in the literature to employ different versions of these models—such as neural networks—for novel COVID-19 modeling. Another reason to investigate novel modeling methods is the fact that correct simulation of COVID-19 in a research region can save money, energy, and time; as a result, the choice of modeling methodology is given a lot of thought when forecasting these important trends [12,13,14,15,16,17,18,19,20]. On the other hand, studies of COVID-19 related to image segmentation have been explored in [12,13,21]. In poorer countries, where the budget for environmental quality evaluation and monitoring is lower than in wealthier ones, modeling approaches are more relevant. According to the Scopus database’s reported literature for 2020–2021, there exists a lot of interest in power system simulation using the feasibility of ML models. The primary keyword occurrence clusters and temporal regional spans across the literature are presented in Figure 1a,b, respectively. Over 2000 articles were included, demonstrating the importance of this topic in terms of COVID-19 modeling. The investigation of new machine learning models capable of solving engineering challenges is always ongoing, and both academics and scientists are interested in the research domain of novel and sophisticated modeling methodologies that can be applied to COVID-19.

The present study makes the following contributions: This is the first research, to the best of the authors’ knowledge, in which an ARIMA model is combined with GLS and deep learning models (random forest (RF) and long-short term memory (LSTM)) to predict CCC under environmental protection knowledge. This research also considers the Economic Community of West African States (ECOWAS), a significant economic grouping. This paper examines the time series characteristics of the CCC using two-unit root tests before modeling, eliminating the risk of relying on a single unit root test. The ARIMA models estimated with ML methods and those estimated with GLS are compared. Despite the fact that ARIMA was used by ArunKumar et al. [7], Alabdulrazzaq et al. [9], and Guleryuz [14], none of these studies applied the GLS, RF, LSTM or EML estimation method and more than one unit root test. This research can help policymakers to analyze hospitalization needs and adopt interventions targeted at flattening the COVID-19 curve.

## 2. Materials and Methods

The current study proposes two different scenarios. The first scenario aimed to model the cumulative COVID-19 cases in four different counties using various classifications of ARIMA models: ARIMA based on automatic routines (AUTOARIMA), ARIMA estimated according to the Box–Jenkins procedure with maximum likelihood method (ARIMAML), ARIMA estimated with the generalized least squares method (ARIMAGLS), and ensembled ARIMAML and ARIMAGLS (ARIMAML-ARIMAGLS). The second scenario employed a novel deep learning model for the estimation of uncertain environmental knowledge regarding forests during the COVID-19 era. For this purpose, the experimental data used in this research were divided, with 70% used for calibration and 30% for the verification phase with validation practices. The model’s results were evaluated using the k-fold cross-validation methodology, which is considered the best way to achieve unbiased model performance prediction with a small data set [7,8]. Although various validation methods can be used, the k-fold cross-validation strategy represents the most practical option for achieving an unbiased goodness-of-fit prediction (for a restricted data set).

**Figure 2:**
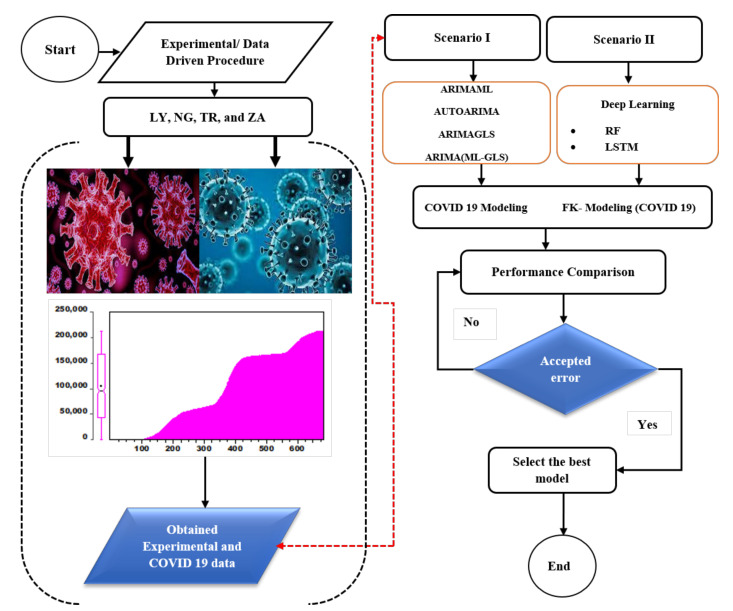
The overall flowchart of the models.

The challenge in determining whether one model outperforms others in reality is the fundamental incentive for using several data-intelligence models. As a result, selecting acceptable models for a specific scenario can be difficult for modelers [22,23]. Only by identifying and selecting several data-driven—and primarily linear—models can this complexity be addressed, despite their shortcomings in handling extremely non-linear and complicated data. Figure 2 presents the flowcharts used in the construction of the current study for scenarios I and II, respectively. Defined in Equation (1), the input data are gathered, pre-processed, and normalized, as shown in the flowchart. The data were normalized before the model was trained, which is commonly carried out to improve the model’s efficiency and accuracy.

(1) $$y=0.05+\left(0.95\times \left(\frac{x-x_{min}}{x_{max}-x_{min}}\right)\right)$$

where *y* represents normalized data, *x* represents measured data, and *x_max_* and *x_min_* represent the measured data’s maximum and minimum values, respectively.

### 2.1. ARIMA Model

**Figure 3:**
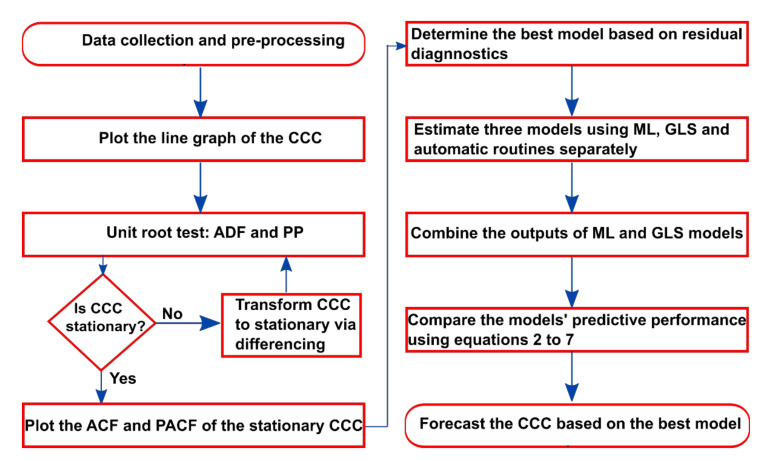
Algorithm for developing the ARIMA models.

Box and Jenkins [24] introduced the ARIMA model concept. Equation (2) represents the ARIMA (p, d, q) model. Autoregressive order, integration order, and moving average order are represented by the letters p, d, and q, respectively. ARIMA is a type of model used in time series forecasting where a collection of observed data from the past are analyzed and used to design a model describing the underlying relationship. This model is further used to predict/extrapolate into the future [20]. A variable’s future value is assumed to be a linear function of several observed data points and random errors [25,26,27,28]. The time series is generated using:

(2) $$\Psi Y_{t}=\alpha_{0}+\Gamma \varepsilon_{t}$$

where $Y_{t}$ is the stationary dependent variable, $\varepsilon_{t}$ is the white noise error term, $\Psi =1-\sum_{i=1}^{p}\alpha_{i}L^{i}$ and $\Gamma =\sum_{j=0}^{q}\beta_{j}L^{j}$, and L is the lag operator defined as $L^{i}Y_{t}=Y_{t-i}$ for i=1,2,…,∞. For more details on ARIMA, see [24]. If $Y_{t}$ is stationary, the series can be modelled as an ARMA (p, q) process, otherwise it has to be modelled as an ARIMA (p, d, q) process. Figure 3 presents the procedures employed by this study in modeling the CCC.

### 2.2. Random Forest (RF)

**Figure 4:**
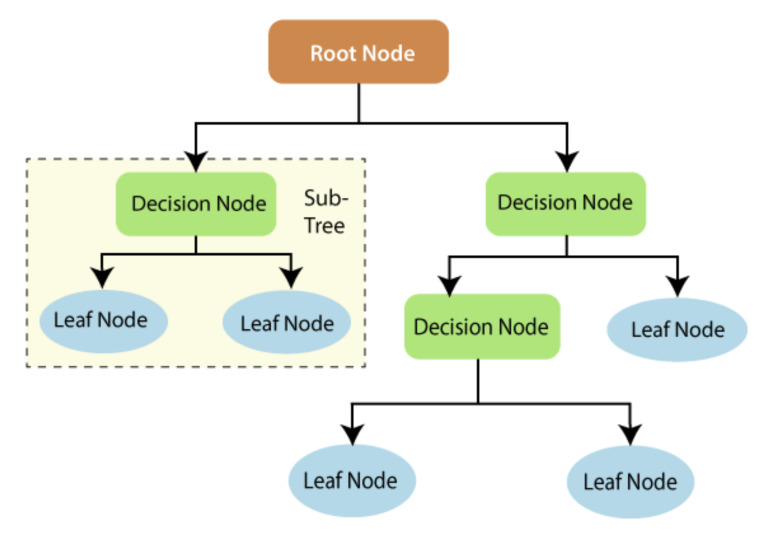
Overall description of RF.

Random forest (RF) is an effective supervised learning technique mainly used for classification and regression problems in machine learning [29]. Breiman, [30] introduced RF as a practical ensemble algorithm which provides an additional non-stationarity layer to the bagging approach [29,30,31,32]. RF fulfils its role by using a random sampling mechanism to generate several decision trees. Generally, forecasts are derived from the mean outputs of these systems, which form a vast ensemble of trees. Bootstrapping or a random selection of inputs are utilized to create the various foundation trees, which is how the decisions are made. Lately, there has been a surge in curiosity regarding RF, which is already used in various applications. The RF architecture used to determine the final features to form the RF tree is presented in Figure 4.

### 2.3. Long Short-Term Memory Neural Network (LSTM)

**Figure 5:**
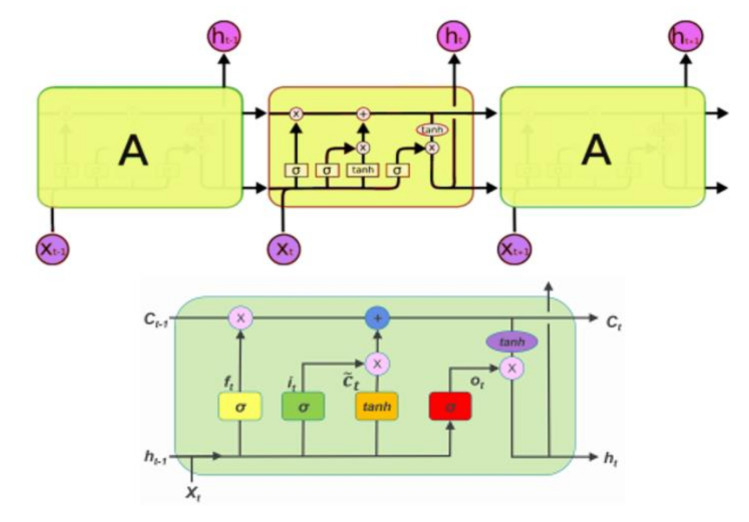
Long short-term memory neural network.

Long short-term memory neural network (LSTM) is a widely used deep learning model in science and engineering. It is capable of analyzing complex and high-dimensional data in a relatively short period with minimal human resources when compared with conventional data collection and analysis [33,34]. The LSTM is a sort of recurrent algorithm that can successfully tackle gradient explosion and gradient disappearance during RNN training while also increasing RNN performance. The LSTM model was created to compensate for the traditional RNN’s inability to memorize sequences of 10 or more characters. The recurrent models are chained iterative approaches that are connected and repeated. The LSTM model, which uses special memory cells to store information, has a chain with an almost identical structure to that of RNN [14,35,36] (see Figure 5).

## 3. Data Processing and Validation

**Figure 6:**
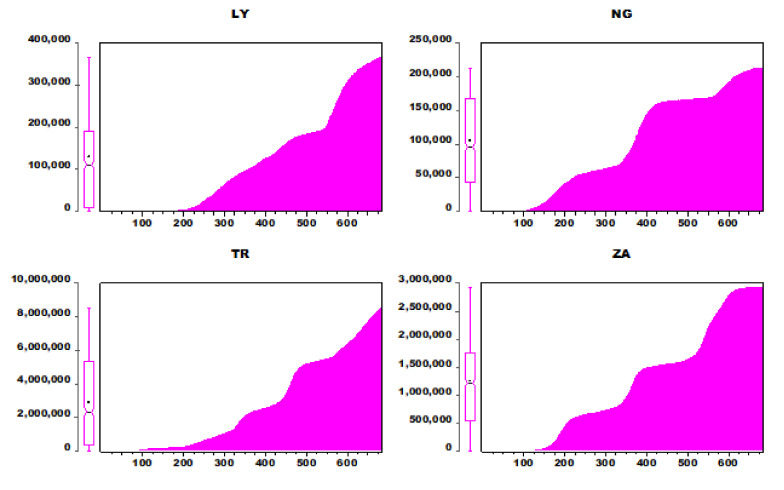
Bar plot of CCC for the four regions.

The following paragraph provides definitions of the variables analyzed in this study. The cumulative total of COVID-19 patients’ daily laboratory records is referred to as the CCC. The data for this study were taken from the World Health Organization’s (WHO) COVID-19 global data database. Each country’s sample runs from the first day a COVID-19 case for the first scenario was recorded in the country through to 1 September 2021. The sample size (N) for each country can be seen in Table 1. Table 1 also shows the CCC’s descriptive data for the four nations (LY, NG, TR, and ZA). The sample size for each country is represented by the number of observations (N). The greater the number of observations, the earlier the country reported the first instance of COVID-19. Accordingly, the first COVID-19 case in the region was discovered in NG, followed by ZA, TR, and then LY. The mean (Y) reflects the CCC’s average value, the median (Y_med_) shows the CCC’s value in the center of the sample for each country, and the standard deviation (σ) represents the CCC’s dispersion from the mean for each country. The earliest number of instances documented for each nation is given by the minimum (Y_min_). For instance, NG’s first COVID-19 record is 5, while LY’s is 1. The maximum value (Y_max_) is the CCC as of 1 September 2021. A histogram of the instances for each country is shown in Figure 6.

**Figure 7:**
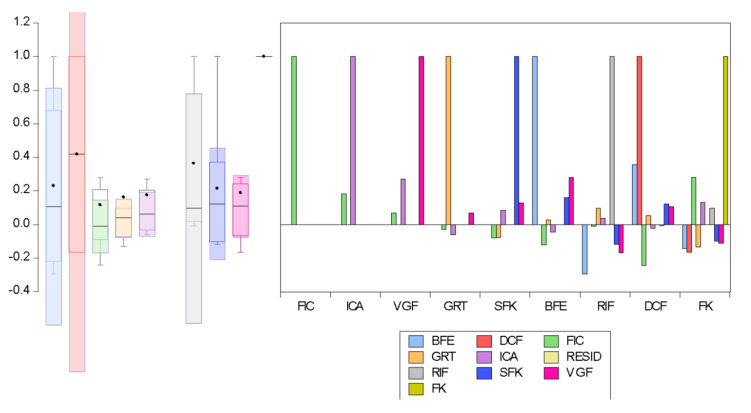
Box-auto correlation analysis between the variables.

For the second scenario, a well-structured questionnaire was developed and subdivided into seven sections with questions and possible responses under subheadings. These included questions on the demographic characteristics of students and questions to evaluate students’ general knowledge regarding forests, forest protection, the importance of forests, poor forest administration, the dangers of deforestation, and how individuals and governments can take responsibility for forests. For exploratory and data-driven analysis, the important variables were selected based on dependency analysis, in which the following variables from the questionnaire were used: forest knowledge (FK), forest importance to the country (FIC), priority for recreational activities (ICA), vital goal of forest (VGF), the government is responsible for taking care of forest problems (GRF), sources of forest knowledge (SFK), benefit of forest protection to man and his environment (BFE), responsibility of individuals to protect the forest in their locality (RIF), and the dangers of cutting forest down (DCF). To understand the effect of COVID-19 on forest knowledge and determine the most dominant parameter, a sensitivity analysis was performed and the results are presented in Figure 7.

The degree to which the relationship between the parameters can be expressed using a linear function and a non-linear function is referred to as sensitivity analysis. The strength of the correlation is not dependent on the direction or sign. A positive coefficient indicates that an increase in the first parameter would correspond to a rise in the second parameter. In contrast, a negative correlation indicates an inverse relationship, in which one parameter increases when the other parameter decreases [37,38].

### Evaluation Criteria

Root mean squared error (RMSE), mean absolute error (MAE), mean absolute percentage error (MAPE), symmetric MAPE (SMAPE), and Theil inequality coefficient (TIC) were the evaluation criteria used in this study. Additionally, the determination coefficient (R^2^) and correlation coefficient (R) were used to assess goodness-of-fit, and one statistical error, the mean squared error (MSE), was used to evaluate models of the second scenario. The above evaluation criteria are presented in Equations (3)–(9).

(3) $$\mathrm{RMSE}=\sqrt{\frac{\sum_{t=T+1}^{T+h}{\left({\hat{y}}_{t}-y_{t}\right)}^{2}}{h}}$$

(4) $$\mathrm{MAE}=\frac{1}{h}\overset{T+h}{\underset{t=T+1}{\sum }}\left|{\hat{y}}_{t}-y_{t}\right|$$

(5) $$\mathrm{MAPE}=\frac{1}{h}\left[\overset{T+h}{\underset{t=T+1}{\sum }}\left|\frac{{\hat{y}}_{t}-y_{t}}{y_{t}}\right|\right]\times 100$$

(6) $$\mathrm{SMAPE}=\frac{1}{h}\left[\overset{T+h}{\underset{t=T+1}{\sum }}\left|\frac{{\hat{y}}_{t}-y_{t}}{{\hat{y}}_{t}+y_{t}}\right|\;\times \;2\right]\;\times \;100$$

(7) $$U_{1}=\sqrt{\frac{\sum_{t=T+1}^{T+h}{\left({\hat{y}}_{t}-y_{t}\right)}^{2}}{h}}\cdot {\left(\sqrt{\frac{\sum_{t=T+1}^{T+h}{\hat{y}}_{t}^{2}}{h}}+\sqrt{\frac{\sum_{t=T+1}^{T+h}y_{t}^{2}}{h}}\right)}^{-1}$$

(8) $$R^{2}=1-\frac{\sum_{j=1}^{N}{\left[{\left(Y\right)}_{\mathrm{obs},j}-{\left(Y\right)}_{\mathrm{com},j}\right]}^{2}}{\sum_{j=1}^{N}{\left[{\left(Y\right)}_{\mathrm{obs},j}-{\bar{\left(Y\right)}}_{\mathrm{obs},j}\right]}^{2}}$$

(9) $$\mathrm{MSE}=\frac{1}{N}\;\sum_{i=1}^{N}{\left(Y_{\mathrm{obsi}}-Y_{\mathrm{comi}}\right)}^{2}$$

(10) $$R=\frac{\sum_{i=1}^{N}\left(Y_{\mathrm{obs}}-{\bar{Y}}_{\mathrm{obs}}\right)\left(Y_{\mathrm{com}}-{\bar{Y}}_{\mathrm{com}}\right)}{\sqrt{\sum_{i=1}^{N}{\left(Y_{\mathrm{obs}}-{\bar{Y}}_{\mathrm{obs}}\right)}^{2}}\sum_{i=1}^{N}{\left(Y_{\mathrm{com}}-{\bar{Y}}_{\mathrm{com}}\right)}^{2}}$$

Equations (3)–(7) include the actual value $y_{t}$, the forecast value ${\hat{y}}_{t}$, the forecast horizon h, and the training/testing sample T.

## 4. Results and Discussion

In this section, the results for both multi-regional COVID-19 modeling and the prediction effect of forest knowledge during the COVID-19 pandemic are analyzed. It is worth mentioning that, to the best of the authors’ knowledge, there is no existing published research that employed this approach. Another motivation for the current research was to conduct a comprehensive bibliographic review of COVID-19 using AI-based models. The results of both analyses are presented in the section below.

### 4.1. Result for Various Type of ARIMA

As stated above, various ARIMA models were employed to analyze and forecast the CCC of four different countries. Prior to modeling, pre-analysis was conducted to determine the reliability of the data. As such, the stationarity of the data was evaluated using formal unit root tests, i.e., augmented Dickey–Fuller (ADF) and Phillips–Perron (PP) tests. These tests have been developed by several studies in the literature. Table 1 presents the results of the unit root test. From the table it can be seen that the CCC values for each country tend to be non-stationary at normal level and become stationary at first difference, excluding TR and ZA which underwent second differences. In addition, ADF revealed that the CCC for TR is I (2) while that of the other countries is I (1). When contradictory findings were found, the PP unit root test took precedence since it can detect near-unit root processes.

ARIMA models have been employed to predict COVID-19-related parameters; for example, Toga et al. [39] applied ARIMA and ANN to forecast COVID-19 prevalence in Turkey. Moreover, ensemble ARIMA has not yet received appropriate attention in the literature. For each of the countries (TR, LY, NG, and ZA), four models were evaluated and their forecast performance was assessed. These four models were: ARIMA with maximum likelihood (ARIMAML), ARIMA with generalized least squares (ARIMAGLS), ARIMA with automatic routines (AUTOARIMA), and ARIMAML with ARIMAGLS (ARIMAML-ARIMAGLS). Before model development, the dominant model selection approach was used, which significantly affects the accuracy of any intelligent computational models. Several input selection approaches including correlation, auto-correlation, and principal components analysis have been reported in the literature but are associated with linearity problems. The minimal value of forecast statistics such as RMSE, MAE, MAPE, SMAPE, Theil U_1_, and Theil U_2_ are presented in Table 2 and Table 3 and were used to assess the performance of the models. This study employed two forecasting techniques: individual building forecasting and ARIMAML and ARIMAGLS ensemble forecasting. The results of ARIMAML and ARIMAGLS are simply averaged. The ensembled model’s output was then analyzed and compared with the output of individual models.

**Figure 8:**
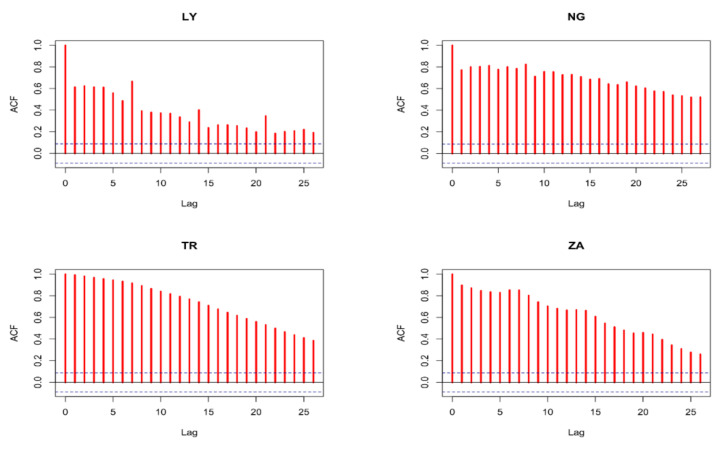
ACF of the first difference of CCC for the four countries.

**Figure 9:**
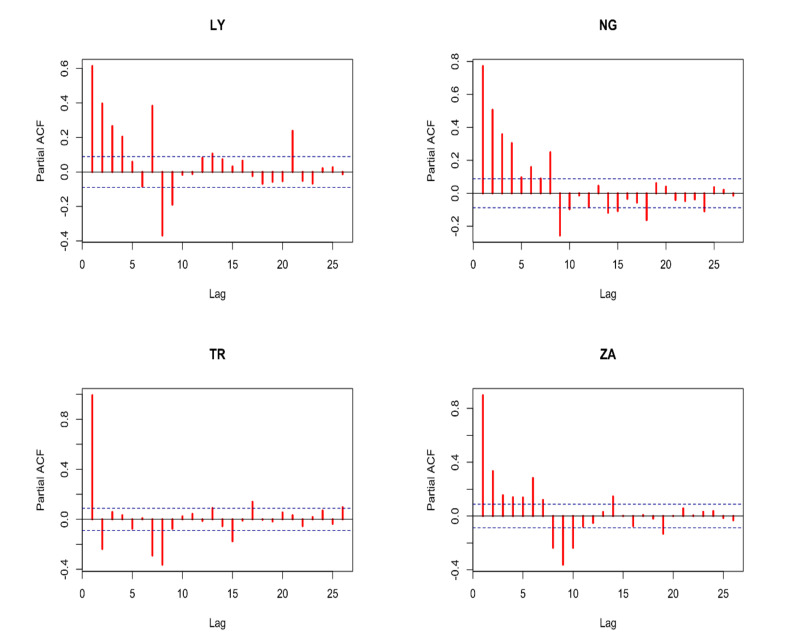
PACF of the first difference of CCC for the four countries.

The ensemble’s principal goal is to create more accurate and dependable estimates than those produced by a single model [40]. It was also confirmed by [41,42,43] that the ensemble technique has numerous advantages over the use of linear modeling methods, including in the initial stage of model selection and in the output of already selected models used for the ensemble. This can reduce the inconsistency in model development because no single model suits and fits all data. The expected performance and accuracy of the model depends on the nature, relationship between variables, uniformity, size, range, etc. of the data, as well as the method used. Because each country’s CCC is integrated of order one, they can be represented by ARIMA (p,1, q) processes. The combination of autoregressive order (p) and moving average order (q) that produced the lowest Schwarz information criteria (SIC) with white-noise mistakes was chosen. Because each country’s CCC is I (1), the initial difference between each CCC was used before estimating. The probable moving average order is determined using the autocorrelation function (ACF), whereas the probable autoregressive order is determined using the partial autocorrelation function (PACF). Figure 8 and Figure 9 show the ACF and PACF graphs, respectively. According to autoregression theory, typical time series data, particularly COVID-19 variables such as CCC, may be forecasted using the lags of the same time series or processes that influence the output variables, as is the case in this study. If the same variable is used, the ACF and PACF may be used to determine the optimal number of lags, whereas the cross-correlation function (CCF) can be utilized if various variables are used.

**Figure 10:**
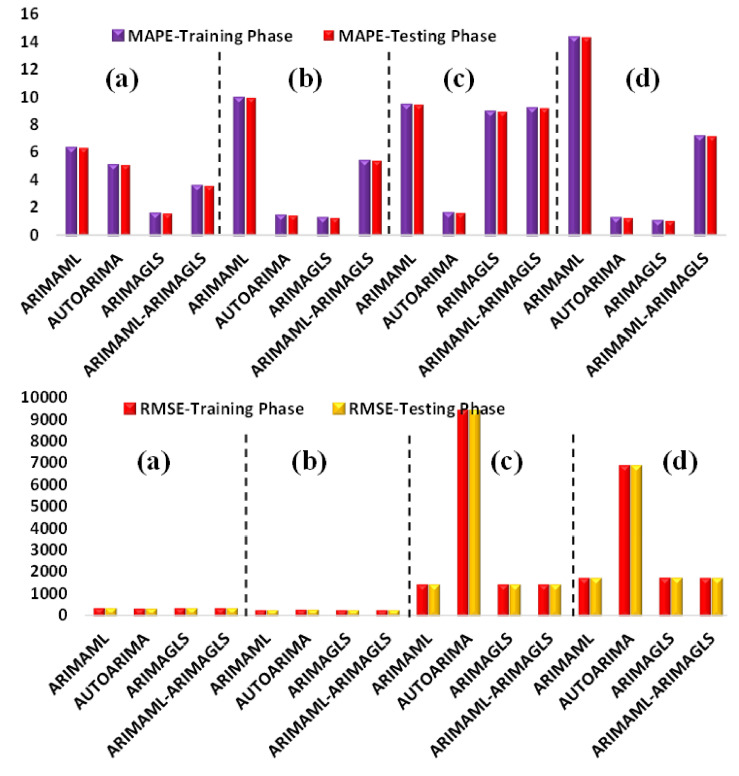
MAPE and RMSE values for: (**a**) LY, (**b**) NG, (**c**) TR, and (**d**) ZA for both training and testing phases.

Understanding the modeling process and data division is quite crucial in any modeling procedure; as such, the modeling forecast for the training phase is presented in Table 3. The results of various performance evaluation matrices, such as RMSE, MAPE, and MAE, are presented for all of the employed models. Based on these results, it can be seen that the lowest RMSE value associated with LY, NG, TR, and ZA is for AUTOARIMA, ARIMA(ML-GLS), ARIMA(ML-GLS), and ARIMAML, respectively. It has been reported in the literature that, for better analysis of model accuracy, different performance indices should be included. MAPE is one such recommended index, and MAPE values between 1–10 is recommended for the best results. The training results indicated that almost all of the model’s performance can be justified by considering the values of MAPE (see Figure 10). According to Figure 10, LY (MAPE = 1.5798), NG (MAPE = 1.2575), TR (MAPE = 1.6228), and ZA (MAPE = 1.0482). ARIMAGLS outperformed the other models for LY, NG, and ZA in terms of MAPE values, while AUTOARIMA was the best model for TR.

**Figure 11:**
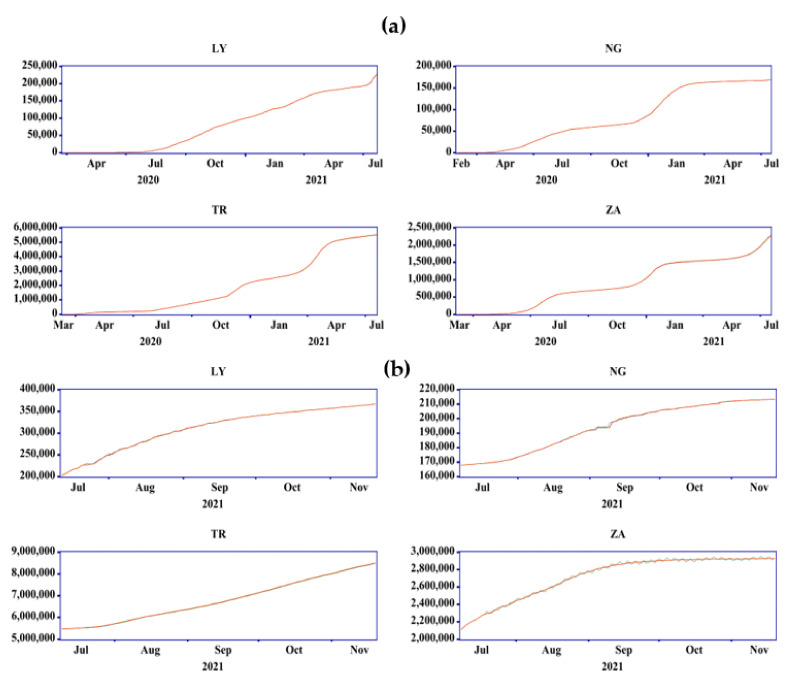
Forecast comparison graph for the: (**a**) training, and (**b**) testing samples.

A quantitative comparison of the results can be conducted using the testing phase sample presented in Table 4. The testing phase represents an essential stage in any modeling in order to validate and generalize the model. The modeling results for CCC show that AUTOARIMA and ARIMAGLS outperformed the other models. It is also worth mentioning that, in most situations, RMSE and Theil U1 agreed on the same model as being the best. It is also worth noting that when other accuracy metrics (MAE, MAPE, SMAPE, and Theil) conflict with RMSE, RMSE takes precedence. Figure 11a,b shows the forecast comparison graphs for the training and testing samples for each nation. The line graphs showing the actual and anticipated levels of CCC are barely distinct. This suggests that using ARIMA to forecast the CCC is valid.

**Figure 12:**
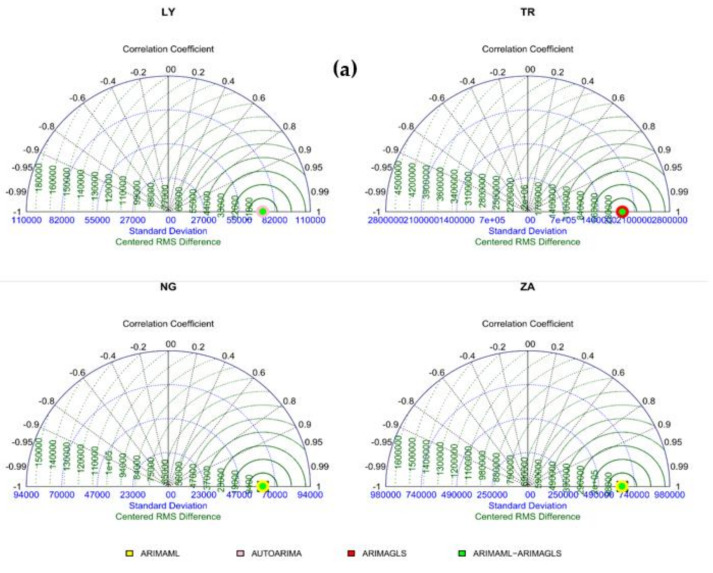

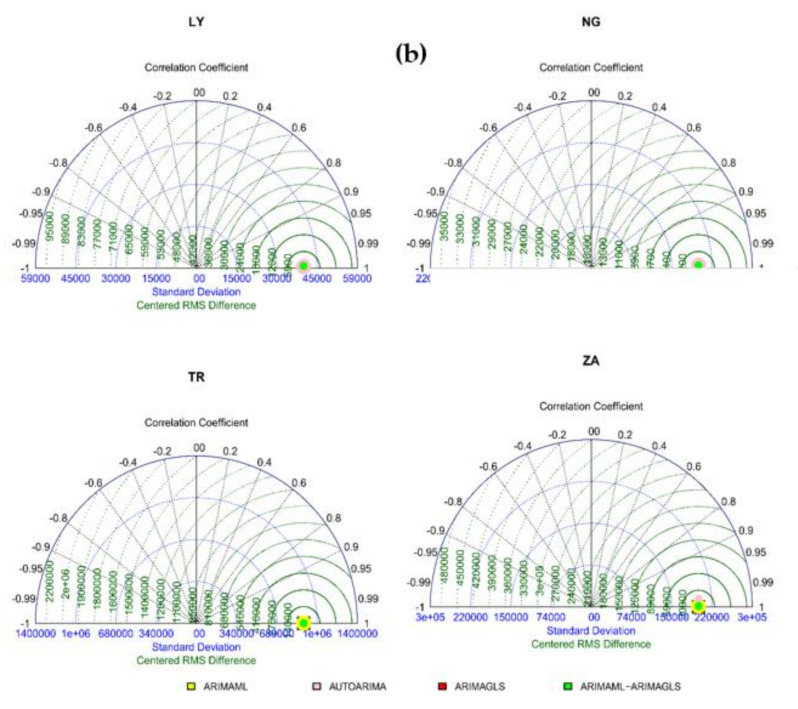
Taylor diagram for the: (**a**) training, and (**b**) testing samples.

**Figure 13:**
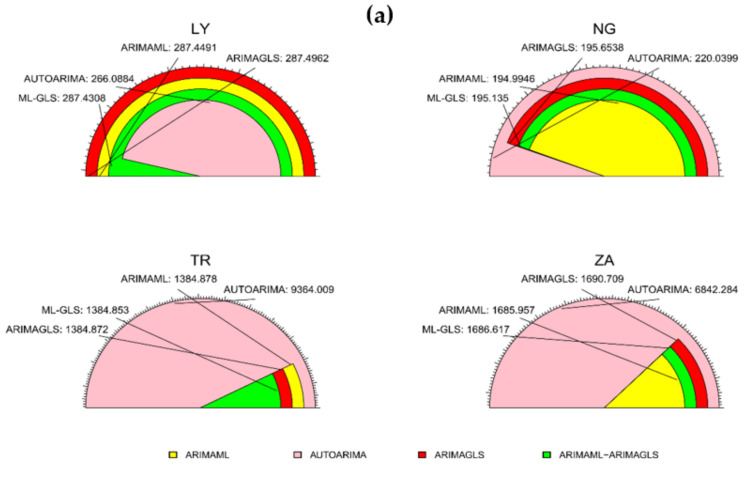

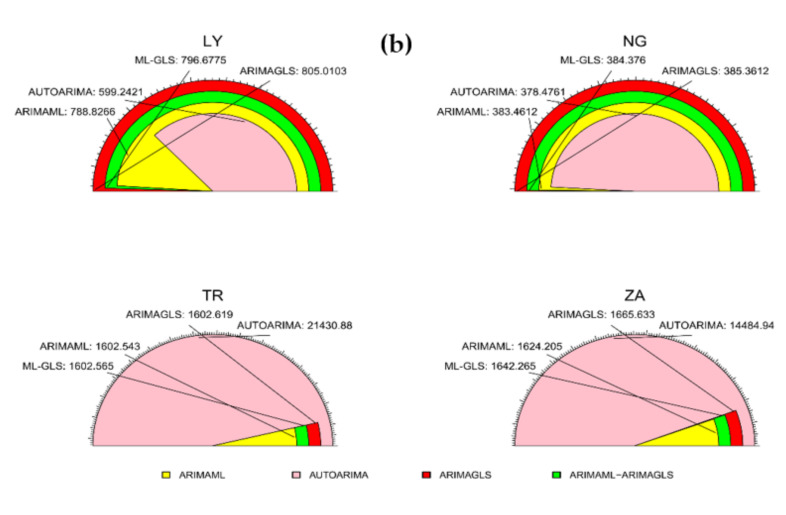
Fan plot of RMSE for the: (**a**) training, and (**b**) testing samples.

The Taylor diagrams for the training and testing samples are shown in Figure 12a,b. The model with the largest dot had the best RMSE. ARIMAML-ARIMAGLS had the best prediction accuracy for seven countries, ARIMAGLS for four countries, and ARIMAML for three countries, as shown in Figure 8. Figure 9 indicates that ARIMAGLS outperforms the other models in predicting accuracy for three countries, whereas ARIMAML outperformed the other models for one country. Table 3 and Table 4 are visually summarized in Figure 8 and Figure 9, respectively. The fan plots for the training and testing samples are shown in Figure 13a,b. The RMSE of the four models are shown on the graph. The greater the model’s prediction performance, the narrower the angle of the sector of the fan plot (see, Figure 13a,b). The performance of four of the best-performing models described in this study was examined in the preceding analysis. The top performing models, according to individual forecast data, are ARIMAGLS and ARIMAML, while combining the two models produces the best prediction accuracy. Because GLS yields inconsistent empirical fit and parameter estimations, the ARIMAGLS model beats other models. Finally, due to the applicability of merging predictions, combining the ARIMAML and ARIMAGLS produces the greatest predicted accuracy in most circumstances when compared with separate models.

### 4.2. Result of Deep Learning Model

For the development of the AI-based models used in the current study, simulations were performed in MATLAB 9.3 (R2020a). Suitable model architecture of both the LSTM and RF models was optimized and selected using trial and error. As reported in the literature [44,45,46], modeling results must satisfy certain evaluation indicators. The outcomes of the simulated models were evaluated using the most utilized performance criteria, including R^2^, MSE, RMSE, and R in both the calibration (70%) and verification (30%) stages. The predicted results were derived from M1 and M2, and the simulated quantitative assessment results are presented in tabular form. Table 5 shows the results of the performance analysis for RF, LSTM, and EML models. It can be seen from the results that all three AI-based models can produce good performance accuracy for the evaluation of FK and management. This is due to the powerful ability of non-linear AI-based models to describe complex systems. Between the two AI-based models (RF and LSTM), SLSTM-M1 emerged as the best combination for FK estimation with values of R^2^ = 0.9393, MSE = 0.0450, and R = 0.9692 in the calibration phase.

**Figure 14:**
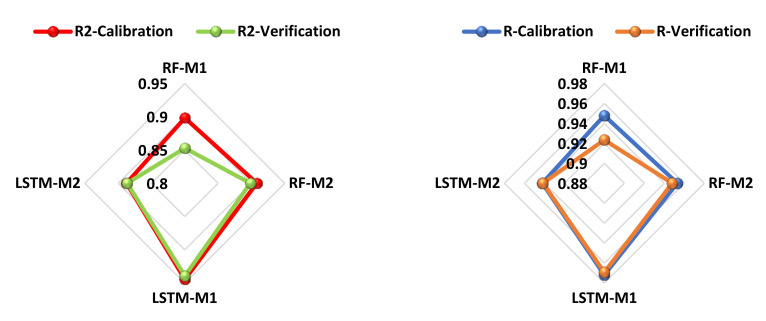
Radar chart for all of the models for R^2^ and R.

**Figure 15:**
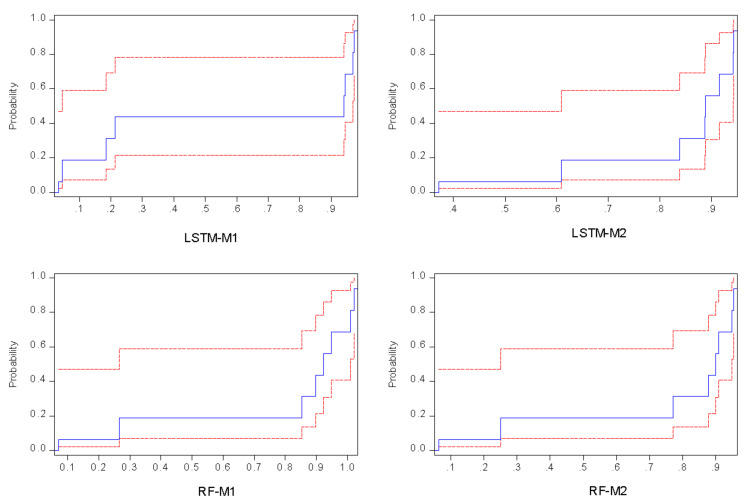
Results of cumulative distribution function for all of the single models.

Further analysis of the results demonstrated that RF-M2 served as the second-best model, follow by RF-M1, and lastly LSTM-M1. The estimation results regarding goodness-of-fit are presented by radar charts (see Figure 14). For the results of single models, it can be concluded that the performance accuracy of the models follows the following order: LSTM-M1 > RF-M2 > ARF-M1 > LSTM-M2. All models achieved good results but cannot serve the purpose of estimation since LSTM is a deep learning model that better captures the non-linear relationship between the variables. Similarly, the profound advantages of deep learning and LSTM include their capability to analyze complex and high-dimensional data in a relatively short period with minimal human resources compared with conventional data collection and analysis. To compare the predictive performance of this study, the R values greater than 0.7 indicated an excellent model. According to [47], R^2^ values greater than 0.8 are satisfactory for any analysis using AI-based models. To further analyze the predictive performance of the model, point-by-point probability plots were generated between the observed and predicted values for the best models, as depicted in Figure 15. From the plots, it can be observed that higher agreement between the observed and predicted values was achieved by LSTM-M1. For this reason, quantitative analysis of the models can be performed using the determination coefficient (R^2^). LSTM-M1 increased the prediction accuracy of the best RF by 4% in the calibration phase and by 2% in the verification phase.

**Figure 16:**
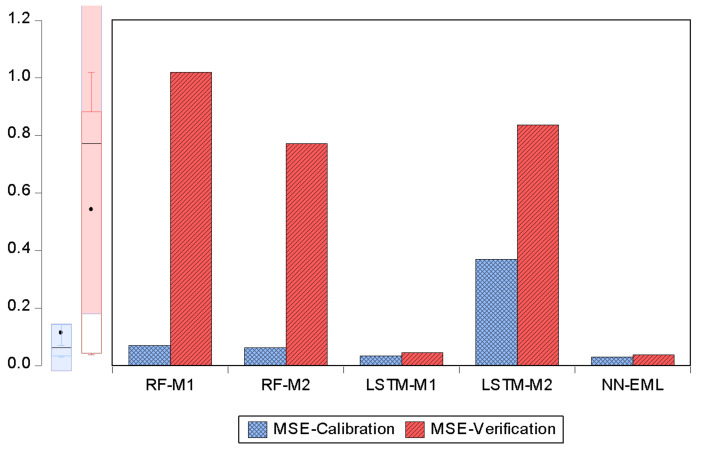
Error performance in term of MSE for all of the models.

The data used in the second scenario were pre-analyzed using a variety of techniques including normalization and reliability tests. The conceptual understanding of each input parameter is critical in assessing the strength of predictive models in soft-computing analysis. As a result, all of the study regions were subjected to stationery and consistency studies utilizing Cronbach’s alpha technique and unit root test. It should be noted that the preliminary examination of a single parameter or input for any time series is extremely important because their forecast accuracy might significantly add to the models’ improved performance. According to Dickey et al. [22], the ADF test is essential for obtaining trustworthy and valid results that ensure the stationarity of all variables. All of the criteria mentioned were met by the experimental data used in this study. The findings demonstrate that the computational modeling methodologies explored have varied levels of appropriateness when considering the evaluation criteria. Furthermore, the aggregate findings showed that EML was the most effective simulation in terms of performance requirements. Though it is impossible to rank the methods according to their acquired precision, the ELM method model had the best forecast accuracy, with a fit to the data of above 97%. The error plots in Figure 16 present a visual comparison of the model combinations with regards to MSE. For the total goodness-of-fit, an error plot depicts the level of agreement between the observed and projected load. The error map clearly reveals that the ELM method model is more accurate than the RF and LSTM models.

The above findings are supported by the capacity of the ELM approach to handle non-linear systems. Unlike RMSE, MSE seems to have a more natural standard measure error and is explicit. It is a model performance measure that is commonly employed in regression analysis. The MAE for a test set is the average of the basic values of all instances in the verification set’s forecast errors. Table 4 also demonstrates its promise in terms of error values. According to the literature, the lowest MSE values suggest the best results and vice versa. The ensemble model’s efficiency can be linked to the hybrid model’s ability to produce more promising outcomes than a single model. For both research and engineering, it is critical to report how dependable AI-based models are.

**Figure 17:**
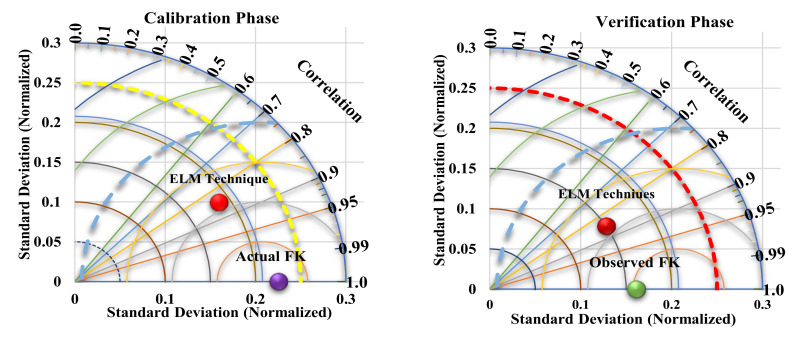
Taylor graphical representations of ensemble models.

The overall judgement between the best single model and ensemble learning is provided using a two-dimensional Taylor diagram, as presented in Figure 17. By considering the actual and estimated values, a Taylor diagram highlights and summarizes several statistical indices such as R, RMSE, and standard deviation [48]. Taylor diagrams can be found in a wide range of fields, including applied and social sciences. Surprisingly, to the best knowledge of the researchers, this is the first study to employ this graph in FK forecasting. In addition, this graphic can be used to compare the internal consistency of different models. As a result, the diagram can be viewed as a collection of polar plot points. A detailed description and discussion of the Taylor diagram can be found in [49]. In the testing phase, the ELM approaches obtained greater goodness of fit with a value of R = 0.98, as shown in Figure 17. These findings show that deep learning and ensemble techniques are capable of capturing complicated non-linear patterns between load demand factors for both training and testing.

## 5. Conclusions

This study estimated and evaluated the forecast performance of four distinct ARIMA models: ARIMAML, ARIMAGLS, AUTOARIMA, and ARIMAML-ARIMAGLS. The models were estimated using CCC time series data for four countries (Brazil, Turkey, Libya, and South Africa). Two sub-samples were employed: 75% for training and 25% for testing. For the training subsample, AUTOARIMA was found to be the best model for Libya, ARIMAGLS for Turkey, and ARIMAML for Nigeria and South Africa. For the testing sample, AUTOARIMA had the best predictive ability for Libya and Nigeria, while ARIMAML was the best for Turkey and South Africa. No evidence was found that ensembling ARIMAML and ARIMAGLS produced the best forecast accuracy in both sub-samples. The results of this study can serve as a reference for modeling the CCC and devising health-related policies.

Nevertheless, for the DL results, AI-based models were developed based on sensitivity analysis to estimate forest knowledge using RF and LSTM models. The performance criteria were evaluated using R^2^, R, MSE, and RMSE. The predictive results demonstrated that AI-based models could predict forest knowledge with less input combination. The results further indicated that all deep learning approach models are capable and satisfactory tools for modeling forest knowledge. Deep learning LSTM-M1 emerged as the best and most reliable estimation model among the AI models analyzed. Although it is difficult to rank the models by their achieved accuracies, the ELM techniques approach showed the best relative prediction accuracy, attaining a goodness of fit greater than 97%. The outcomes also suggested the development of AI-based models in this field. Other non-linear models and optimization techniques should be employed, such as non-linear ensemble techniques (NET), gaussian process regression models (GPRM), gradient boasting (GB), extreme learning machines (ELM), genetic algorithms (GA), emerging optimization (EO), and kernel models (KM) to improve the estimation accuracy. It is also suggested to expand these techniques to other geo-environmental locations across the globe. This is in line with Areepong and Sunthornwat, [50] who concluded that future research should focus on estimating the maximum number of visitors that can enter a country while maintaining control of the number of COVID-19 cases. It may also be of interest to investigate the use of both forecasting models to anticipate and assess the spread of COVID-19 in other nations.

## Figures and Tables

**Table 1:** Descriptive statistics of the variables.

	$Y^{-}$	$Y_{med}$	$Y_{max}$	$Y_{min}$	σ	N
LY	129,333.3	111,124	366,789	1	116,504.4	605
NG	104,581.5	95,934	213,464	5	72,519.22	631
TR	2,906,611	2,355,839	8,503,220	1	2,635,359	619
ZA	1,252,298	1,231,597	2,927,499	5	960,752.1	625

**Table 2:** ADF and PP unit root test results.

Variables	None	Constant	Constant and Trend	None	Constant	Constant and Trend	Decision
LY	2.676	1.594	−1.986	−2.291 ***	−3.933 ***	−4.527 ***	I(1)
NG	1.244	−0.705	−2.225	−2.179 ***	−3.058 ***	−3.041 ***	I(1)
TR	0.931	0.311	−2.582	−0.89	−1.865	−2.167	I(2)
ZA	0.929	−0.444	−3.166	−1.72	−2.368	−2.277	I(2)
LY	7.204	3.413	−1.797	−10.572 ***	−17.217 ***	−20.344 ***	I(1)
NG	3.755	−0.116	−1.627	−11.264 ***	−17.258 ***	−17.263 ***	I(1)
TR	7.839	3.865	−1.414	−0.907	−1.882	−2.143	I(2)
ZA	4.329	0.882	−2.221	−4.091 ***	−4.112 ***	−4.567 ***	I(1)

*** signifies regrectionof null hypothesis at 1% level of significance.

**Table 3:** Evaluation results for the training sample.

Models	RMSE	MAE	MAPE	SMAPE	Theil U1	Theil U2
ARIMAML	287.4491	171.1810	6.329595	4.651772	0.001302	4.456228
AUTOARIMA	266.0884	158.2471	5.097415	4.000233	0.001204	3.065781
ARIMAGLS	287.4962	170.3428	1.579800	1.613471	0.001302	0.904637
ARIMAML-ARIMAGLS	287.4308	170.5967	3.587180	3.010201	0.001302	2.431895
ARIMAML	194.9946	107.5219	9.913560	5.122160	0.000946	4.291490
AUTOARIMA	220.0399	124.1118	1.442778	1.494742	0.001067	0.834548
ARIMAGLS	195.6538	106.9622	1.257531	1.378221	0.000949	0.900122
ARIMAML-ARIMAGLS	195.1350	107.0657	5.410406	3.742184	0.000946	2.199975
ARIMAML	1384.8780	667.1446	9.421745	1.801850	0.000259	6.971105
AUTOARIMA	9364.0090	6847.1200	1.622860	1.533954	0.001748	0.715107
ARIMAGLS	1384.8720	666.1256	8.916807	1.775690	0.000259	6.619425
ARIMAML-ARIMAGLS	1384.8530	666.6342	9.169276	1.788919	0.000259	6.795260
ARIMAML	1685.9570	818.2752	14.266580	3.603760	0.000768	13.075140
AUTOARIMA	6842.2840	4613.7460	1.256974	1.241767	0.003113	0.538986
ARIMAGLS	1690.7090	819.5312	1.048277	1.105426	0.000770	0.673285
ARIMAML-ARIMAGLS	1686.6170	814.9397	7.180849	2.674733	0.000768	6.467583

**Table 4:** Evaluation results for the testing sample.

Models	RMSE	MAE	MAPE	SMAPE	Theil U1	Theil U2
ARIMAML	287.4491	171.1810	6.329595	4.651772	0.001302	4.456228
AUTOARIMA	266.0884	158.2471	5.097415	4.000233	0.001204	3.065781
ARIMAGLS	287.4962	170.3428	1.579800	1.613471	0.001302	0.904637
ARIMAML-ARIMAGLS	287.4308	170.5967	3.587180	3.010201	0.001302	2.431895
ARIMAML	194.9946	107.5219	9.913560	5.122160	0.000946	4.291490
AUTOARIMA	220.0399	124.1118	1.442778	1.494742	0.001067	0.834548
ARIMAGLS	195.6538	106.9622	1.257531	1.378221	0.000949	0.900122
ARIMAML-ARIMAGLS	195.1350	107.0657	5.410406	3.742184	0.000946	2.199975
ARIMAML	1384.8780	667.1446	9.421745	1.801850	0.000259	6.971105
AUTOARIMA	9364.0090	6847.1200	1.622860	1.533954	0.001748	0.715107
ARIMAGLS	1384.8720	666.1256	8.916807	1.775690	0.000259	6.619425
ARIMAML-ARIMAGLS	1384.8530	666.6342	9.169276	1.788919	0.000259	6.795260
ARIMAML	1685.9570	818.2752	14.266580	3.603760	0.000768	13.075140
AUTOARIMA	6842.2840	4613.7460	1.256974	1.241767	0.003113	0.538986
ARIMAGLS	1690.7090	819.5312	1.048277	1.105426	0.000770	0.673285
ARIMAML-ARIMAGLS	1686.6170	814.9397	7.180849	2.674733	0.000768	6.467583

**Table 5:** Results and performance analysis of the models.

		Calibration Phase				Verification Phase		
Models	R^2^	MSE	R	RMSE	R^2^	MSE	R	RMSE
RF-M1	0.8982	0.0705	0.9477	0.2655	0.8526	1.0205	0.9234	1.0102
RF-M2	0.9082	0.0626	0.9530	0.2502	0.8985	0.7715	0.9479	0.8784
LSTM-M1	0.9447	0.0336	0.9720	0.1833	0.9393	0.0450	0.9692	0.2121
LSTM-M2	0.8876	0.3705	0.9421	0.6087	0.8864	0.8374	0.9415	0.9151
NN-EML	0.9776	0.0305	0.9881	0.1746	0.9694	0.0374	0.9845	0.1933

## Data Availability

All supporting information is accessible upon request from the editor-in-chief. Other data that support the conclusions of this study may be found in the World Health Organization’s (WHO) COVID-19 database, which can be accessed at the following site: https://covid19.who.int/info, accessed on 10 November 2021. In addition, the data was accessed on 30 November 2021.
